# Supplementary material for: Interactome profiling of Crimean-Congo hemorrhagic fever virus glycoproteins
Source: Nat Commun. 2023 Nov 14;14:7365. doi: 10.1038/s41467-023-43206-1 (PMC10646030; doi:10.1038/s41467-023-43206-1)
Supplement: Supplementary file 1 — Supplementary Information [file 41467_2023_43206_MOESM1_ESM.pdf]

Supplementary information

Interactome profiling of Crimean-Congo hemorrhagic fever virus glycoproteins

Shiyu Dai[a,b,#], Yuan-Qin Min[a,c,#], Qi Li[a,d], Kuan Feng[a,c], Zhenyu Jiang[a,d], Zhiying Wang[a], Cunhuan Zhang[a], Fuli Ren[a,], Yaohui Fang[a,d], Jingyuan Zhang[a,d], Qiong Zhu[a,c], Manli Wang[a,c], Hualin Wang[a,c,*], Fei Deng[a,c,*], Yun-Jia Ning[a,c,e,*]

[a]Key Laboratory of Virology and Biosafety and National Virus Resource Center, Wuhan Institute of Virology, Chinese Academy of Sciences, Wuhan 430071/430207, China; [b]Department of Cardiovascular Surgery of the First Affiliated Hospital & Institute for Cardiovascular Science, Suzhou Medical College, Soochow University, Suzhou 215006, China; [c]State Key Laboratory of Virology and Center for Biosafety Mega-Science, Chinese Academy of Sciences, Wuhan 430071/430207, China; [d]University of Chinese Academy of Sciences, Beijing 101408, China; [e]Hubei Jiangxia Laboratory, 430200, Wuhan, China

[#]Co-first authors.

*Corresponding authors.

This PDF file includes:

Supplementary figures S1–S7;

Supplementary tables S1–S3.

Supplementary Figures

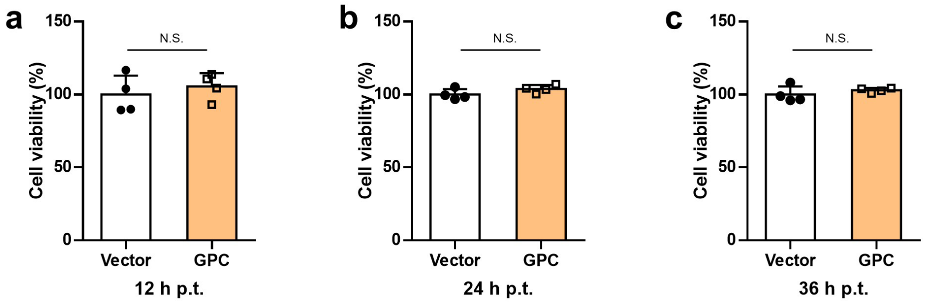

Supplementary Figure. S1: Effect of CCHFV GPC on cell viability. HEK293T cells were transfected with the GPC expression plasmid or pCAGGS vector as the control. At indicated time, cell viability was measured using the Cell Counting Kit-8 (CCK8) assay as described in Methods. Relative cell viability rates were calculated by normalization to the control (vector) groups. Values represent means ± SD (n = 4 biologically independent samples). Two-tailed unpaired *t*-test was performed. N.S., not significant. Source data are provided as a Source Data file.

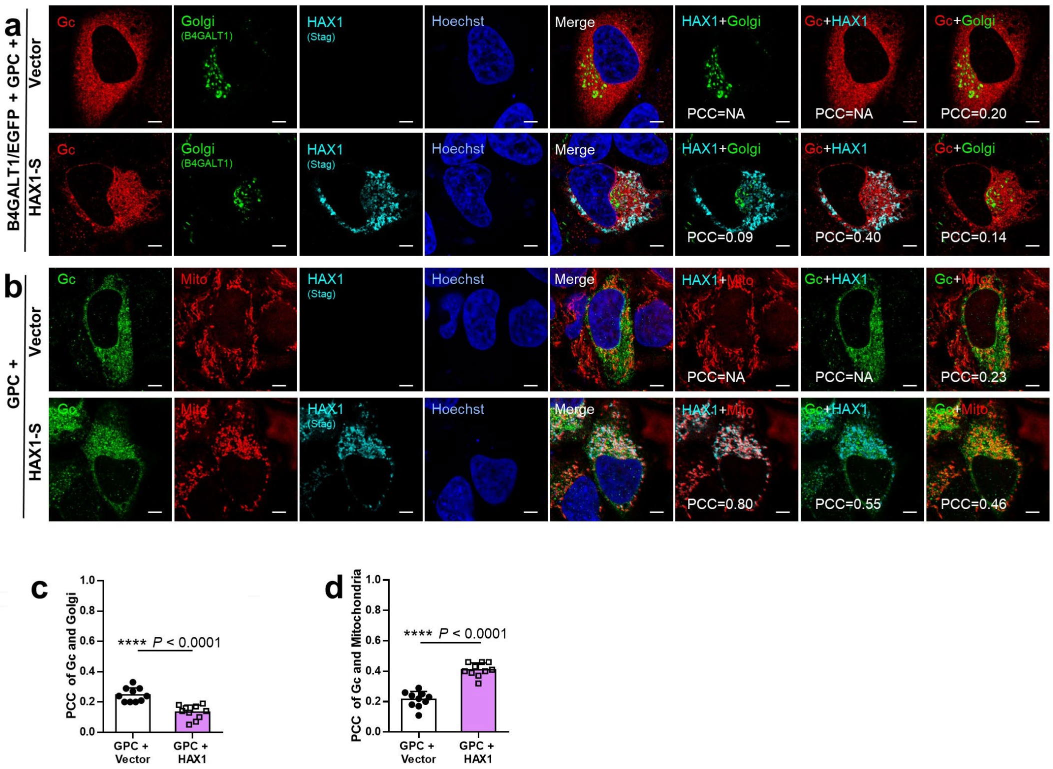

Supplementary Figure. S2: Effects of HAX1 on subcellular localization of Gc. a Effect of HAX1 on the localization of Gc to the Golgi apparatus. HeLa cells were transfected with plasmids encoding the indicated proteins, CCHFV GPC, B4GALT1 fused with EGFP (B4GALT1/EGFP, as the Golgi marker), and HAX1 fused with Stag (HAX1-S), or the control vector. At 36 h p.t., cells were fixed and the localization of Gc (red), HAX1 (cyan), and B4GALT1/EGFP (green) were visualized by confocal microscopy after immunofluorescence staining. Nuclei stained with Hoechst are shown in blue. Representative images are shown. PCC, Pearson's correlation coefficient. **b** Effect of HAX1 on the localization of Gc to mitochondria. HeLa cells were transfected with plasmids encoding the indicated proteins or the control vector. At 36 h p.t., mitochondria were stained with MitoTracker red CMXRos prior to fixation, followed by detection of Gc (green), HAX1 (cyan), and mitochondria (red) by IFA and confocal microscopy. **c** Co-localization analysis of Gc and Golgi apparatus. **d** Co-localization analysis of Gc and mitochondria. Colocalization was assessed by PCC using the *coloc*2-plugin of the extended ImageJ version Fiji as described in Methods. Bars, 5 μm. Two-tailed unpaired *t*-test was performed. Data are presented as means ± SD (n = 10 cells). ****, $P < 0.0001$. These experiments were repeated for three times with similar results (**a**-**b**). Source data (**c**-**d**) are provided as a Source Data file.

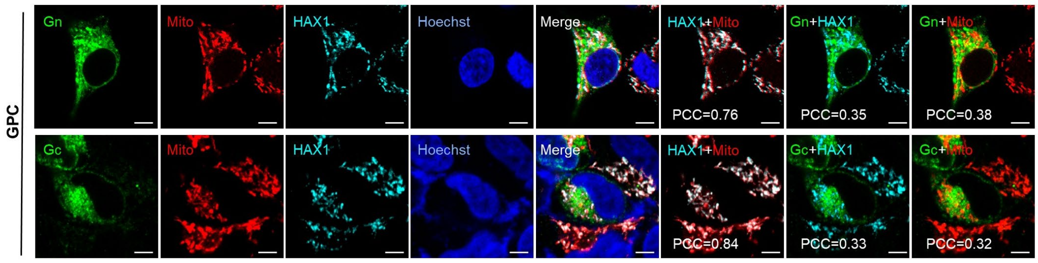

Supplementary Figure. S3: Mitochondrial localization of Gn/Gc in the context of endogenous HAX1 expression. Cells transfected with the GPC expression plasmid were stained with MitoTracker red CMXRos prior to fixation at 36 h p.t. for visualization of mitochondria (red), followed by detection of Gn/Gc (green), and endogenous HAX1 (cyan) by IFA and confocal microscopy. Bars, 5 μm. Data are representative of three independent experiments with similar results.

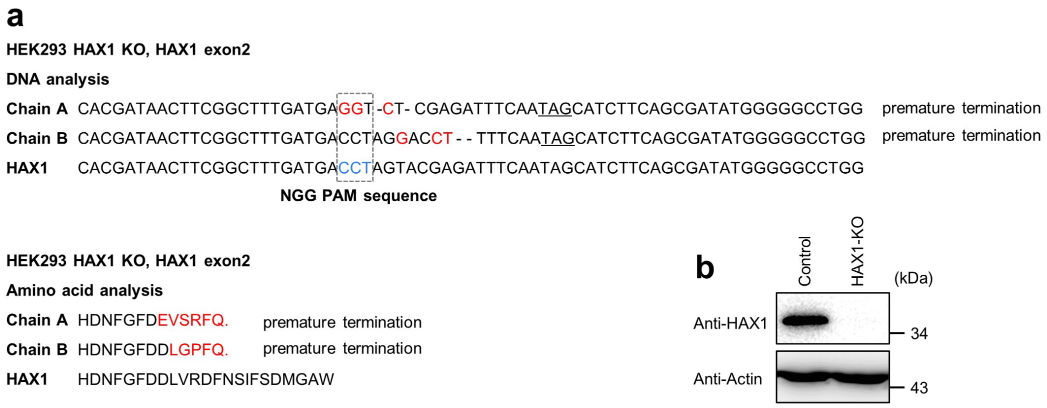

Supplementary Figure. S4: Validation of HAX1-knockout (KO) HEK293 cells generated by CRISPR-Cas9. a DNA sequencing of HAX1-KO HEK293 cells. Region encompassing the modified region (second exon of HAX1) was amplified by PCR and sequenced. The PAM sequences were indicated in dashed boxes. Resulting protein sequences were shown below the aligned DNA sequences. **b** Western blot analysis of HAX1-KO HEK293 cells. The generated cell line was subjected to Western blot analysis with the indicated antibodies to validate the deletion of HAX1 expression. The experiment was repeated for three times with similar results (**b**). See also the Methods for experimental details. Source data are provided as a Source Data file.

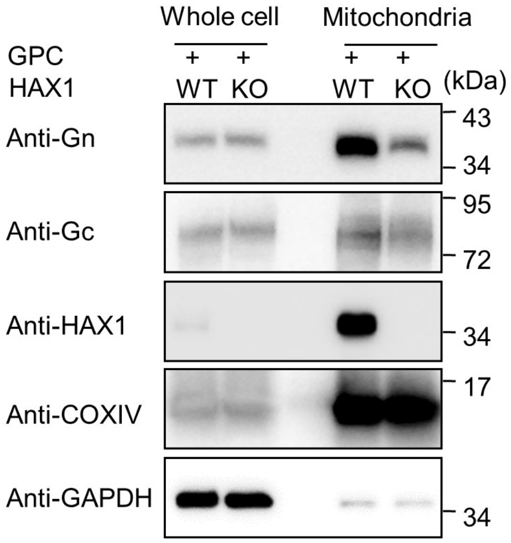

Supplementary Figure. S5: Mitochondrion fractionation assays showing Gn/Gc enrichments to mitochondria and the ablation by HAX1 knockout. HAX1 knockout (KO) or wild-type (WT) HEK293 cells were transfected with the GPC expression plasmid and Gn/Gc enrichments in mitochondrial fractions were detected by Western blot analysis as described in Fig. 5**e**. COXIV was used as the marker for mitochondria. Data are representative of three independent experiments with similar results. Related to Fig. 5**e**. Source data are provided as a Source Data file.

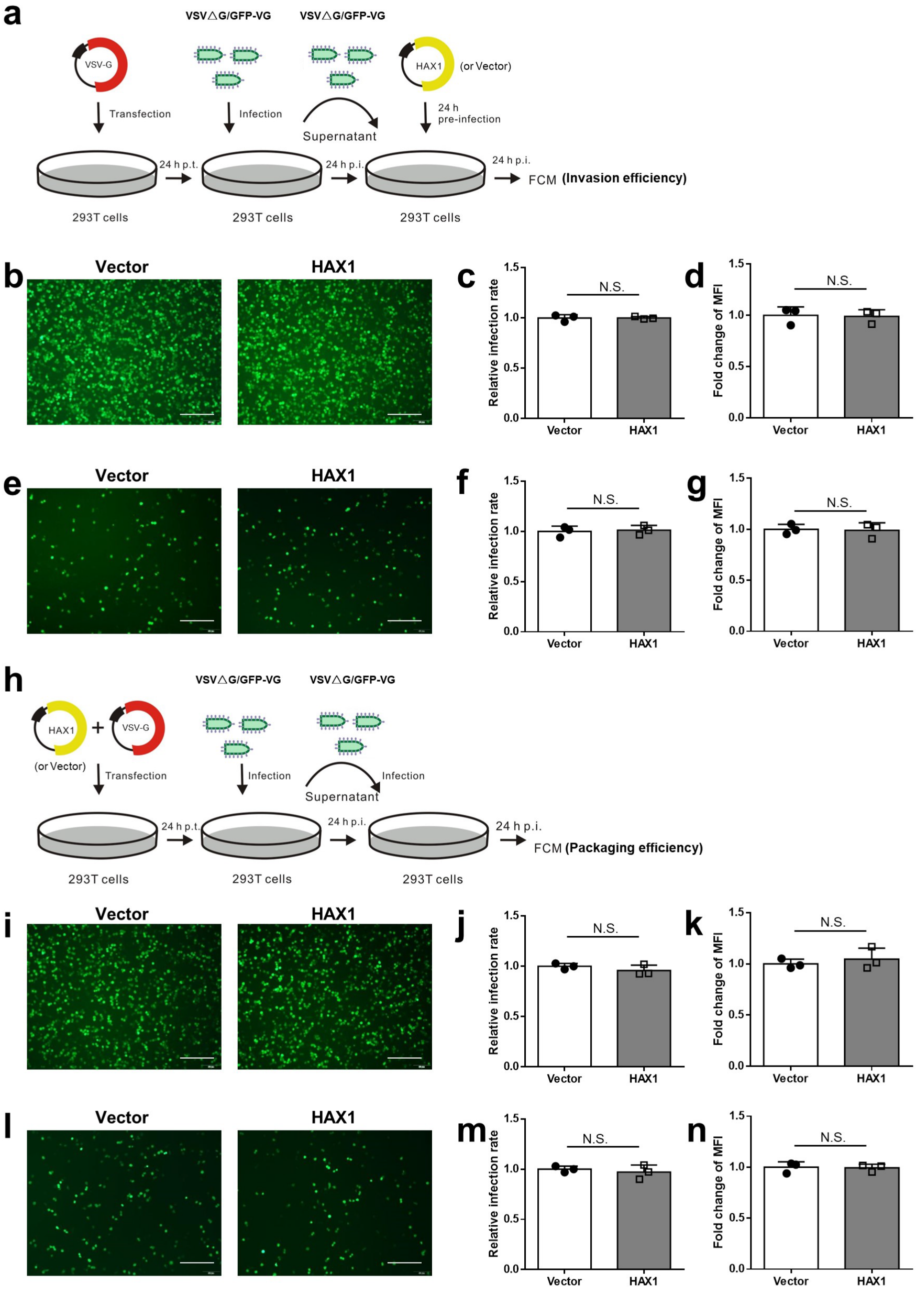

Supplementary Figure. S6: Neither VSV G-mediated packaging nor invasion is significantly affected by HAX1. Effects of HAX1 on VSV G-mediated invasion (**a-g**) and packaging (**h-n**) were assessed with the pseudovirus system similarly to Fig. 7, except that a VSV G expression plasmid was used to replace the CCHFV GP plasmid for transfection and correspondingly the transduction with VSV△G/GFP-VG was analyzed by fluorescence microscopy (**b** and **i**) and flow cytometry (**c**, **d**, **j**, and **k**). As the packaging efficiency by VSV G is higher than that by CCHFV GP, the VSV G-pseudotyped viruses with 5-fold dilution were also used for the evaluation and consistent results were obtained (**e-g** and **l-n**). Bars, 20 μm. VSV△G/GFP-VG, GFP-expressing pseudotyped VSV bearing VSV G. Values represent means ± SD (n = 3 biologically independent samples). Two-tailed unpaired *t*-test was performed. N.S., not significant. Source data are provided as a Source Data file.

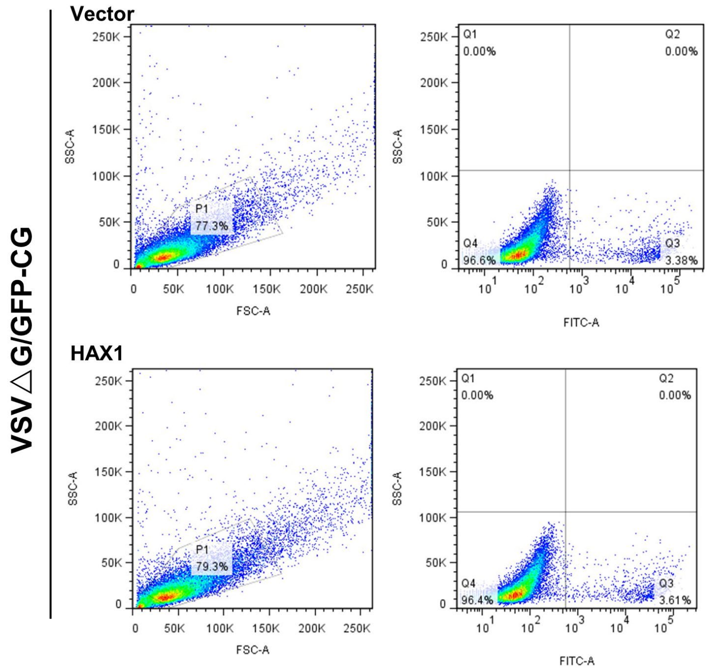

Supplementary Figure. S7: Representative pseudo-color dot plots exemplifying gating strategy. Representative pseudo-color dot plots corresponding to the experiments in Fig. 7a-7d were shown with the gating strategy. Gating based FSC-H vs SSC-H was first conducted for exclusion of cell debris and adhesion. Then, the cell populations selected was gated by FITC (EGFP) signals.

Supplementary Table S1. CCHFV glycoprotein Gn-interacting host proteins

Accession	Name	Description	log2 FC	P-value
Q14974	KPNB1	Importin subunit beta-1	6.196325	0.00009857
D6RCE2	TTC37	Tetratricopeptide repeat protein 37	4.904325	0.0177
E7ES19	THBS4	Thrombospondin-4	6.46545	0.02251
O43402	EMC8	ER membrane protein complex subunit 8	6.011975	0.02431
Q13885	TUBB2A	Tubulin beta-2A chain	5.6584	0.000001483
P06576	ATP5F1B/ATP5B	ATP synthase subunit beta, mitochondrial	3.278325	0.0004095
P00403	MT-CO2	Cytochrome c oxidase subunit 2	4.866	0.00001586
F5H608	ATP5PD/ATP5H	ATP synthase subunit d, mitochondrial	2.6432	0.03129
P11021	HSPA5	Endoplasmic reticulum chaperone BiP	4.76295	0.0007034
P25705	ATP5F1A/ATP5A1	ATP synthase subunit alpha, mitochondrial	3.6983	0.009652
P30825	SLC7A1	High affinity cationic amino acid transporter 1	4.44965	0.0000704
P30049	ATP5F1D/ATP5D	ATP synthase subunit delta, mitochondrial	2.553525	0.01617
O00264	PGRMC1	Membrane-associated progesterone receptor component 1	4.2739	0.002853
P05023	ATP1A1	Sodium/potassium-transporting ATPase subunit alpha-1	3.9041	0.003898
A0A1W2PQM2	TUBA1C	Tubulin alpha-1C chain	3.775975	0.003207
A6NLM8	SSR4	Translocon-associated protein subunit delta	3.74445	0.001914
E9PIQ7	HAX1	HCLS1-associated protein X-1	3.61435	0.00003402
H3BNV7	CIAO2B/FAM96B	Cytosolic iron-sulfur assembly component 2B (Fragment)	3.590725	0.001154
O95399	UTS2	Urotensin-2	3.579775	0.000013
P35232	PHB	Prohibitin	2.69985	0.004816
F5GZQ3	HADHB	Trifunctional enzyme subunit beta, mitochondrial	2.541025	0.000002816
P16615	ATP2A2	Sarcoplasmic/endoplasmic reticulum calcium ATPase 2	3.30845	0.0001057
P04792	HSPB1	Heat shock protein beta-1	3.265975	0.001318
B1AHE3	ATXN10	Ataxin-10	2.768075	0.02805
P50402	EMD	Emerin	2.63135	0.0009484
R4GMX5	BSG	Basigin (Fragment)	2.460275	0.002916
Q5JP53	TUBB	Tubulin beta chain	2.4432	0.00005076
A0A286YFM8	PHGDH	D-3-phosphoglycerate dehydrogenase (Fragment)	2.440575	0.00008864
O00483	NDUFA4	Cytochrome c oxidase subunit NDUFA4	2.402625	0.001018
P68371	TUBB4B	Tubulin beta-4B chain	2.2713	0.00003059
A8MXP8	RCN2	Reticulocalbin-2	2.265825	0.04479
Q92504	SLC39A7	Zinc transporter SLC39A7	1.88175	0.00008605
P11142	HSPA8	Heat shock cognate 71 kDa protein	1.67915	0.002853
P0DMV9	HSPA1B	Heat shock 70 kDa protein 1B	1.285825	0.002327

Two-tailed, equal-sample variance Student's *t* test was carried out to calculate the *p*-Values.

Supplementary Table S2. CCHFV glycoprotein Gc-interacting host proteins

Accession	Name	Description	log2 FC	P-value
Q14974	KPNB1	Importin subunit beta-1	7.14815	0.00003053
D6RCE2	TTC37	Tetratricopeptide repeat protein 37 (Fragment)	6.918125	0.000002255
A0A087WXU8	DDX24	ATP-dependent RNA helicase DDX24	6.273825	0.02926
O43402	EMC8	ER membrane protein complex subunit 8	6.063825	0.00001242
Q13885	TUBB2A	Tubulin beta-2A chain	4.109475	0.01268
P06576	ATP5F1B/ATP5B	ATP synthase subunit beta, mitochondrial	5.1908	0.00001814
P00403	MT-CO2	Cytochrome c oxidase subunit 2	3.683475	0.00005496
F5H608	ATP5PD/ATP5H	ATP synthase subunit d, mitochondrial	4.823925	0.00004772
A0A2R8Y4F5	HADHA	Trifunctional enzyme subunit alpha, mitochondrial	4.79135	0.000004588
P11021	HSPA5	Endoplasmic reticulum chaperone BiP	3.184275	0.006451
P25705	ATP5F1A/ATP5A1	ATP synthase subunit alpha, mitochondrial	4.666675	0.003483
P30049	ATP5F1D/ATP5D	ATP synthase subunit delta, mitochondrial	4.41145	0.0004518
O00264	PGRMC1	Membrane-associated progesterone receptor component 1	2.64095	0.02613
G3V5P0	KTN1	Kinectin (Fragment)	4.236375	0.01286
A0A1W2PQM2	TUBA1C	Tubulin alpha-1C chain	2.596725	0.01308
A6NLM8	SSR4	Translocon-associated protein subunit delta	3.281475	0.002932
E9PIQ7	HAX1	HCLS1-associated protein X-1	2.93105	0.00004209
P35232	PHB	Prohibitin	3.466575	0.0007525
F5GZQ3	HADHB	Trifunctional enzyme subunit beta, mitochondrial	3.387225	0.0000007185
P04792	HSPB1	Heat shock protein beta-1	2.658525	0.001605
P04843	RPN1	Dolichyl-diphosphooligosaccharide--protein glycosyltransferase subunit 1	3.03805	0.004595
E9PMW7	EEF1D	Elongation factor 1-delta	2.9371	0.00009355
K7ENK9	VAMP2	Vesicle-associated membrane protein 2	2.778325	0.007085
O43819	SCO2	Protein SCO2 homolog, mitochondrial	2.733475	0.0001867
P20674	COX5A	Cytochrome c oxidase subunit 5A, mitochondrial	2.69055	0.04128
P50402	EMD	Emerin	1.866075	0.005179
Q5JP53	TUBB	Tubulin beta chain	1.604	0.0001431
A0A286YFM8	PHGDH	D-3-phosphoglycerate dehydrogenase (Fragment)	1.403425	0.003597
J3QS39	UBB	Polyubiquitin-B (Fragment)	2.287925	0.02821
P68371	TUBB4B	Tubulin beta-4B chain	1.448125	0.00154
P11142	HSPA8	Heat shock cognate 71 kDa protein	1.5011	0.002031
C9K0U8	SSBP1	Single-stranded DNA-binding protein, mitochondrial (Fragment)	1.27505	0.009058
P23284	PPIB	Peptidyl-prolyl cis-trans isomerase B	1.235975	0.003324

Two-tailed, equal-sample variance Student's *t* test was carried out to calculate the *p*-Values.

Supplementary Table S3. Oligonucleotides used in this study

Oligonucleotide	Sequence (5' to 3')	Purpose
**Primer**		
**CCHFV S-segment-Forward**	TCGCCGATTACCAACAGGCTG	CCHFV S-segment, qPCR primers
**CCHFV S-segment-Reverse**	CCTGCACCACTCCACATGTTC	
**Human-HAX1-Forward**	CGAGATGAAGATGATGATGAG	Human HAX1, qPCR primers
**Human-HAX1-Reverse**	GTGCTGAGGACTATGGAA	
**Human-β-Actin-Forward**	CATCCGTAAAGACCTCTATGCCAAC	Human β-Actin, qPCR primers
**Human-β-Actin-Reverse**	ATGGAGCCACCGATCCACA	
**Mouse-HAX1-Forward**	CGAGGCTTTTTCGGCTTTCC	Mouse HAX1, qPCR primers
**Mouse-HAX1-Reverse**	GCATAGCTCTCTCGACCCCA	
**Mouse-GAPDH-Forward**	TCTGGAAAGCTGTGGCGTG	Mouse GAPDH, qPCR primers
**Mouse-GAPDH-Reverse**	CCAGTGAGCTTCCCGTTCAG	
**Target sequence**		
**sh human-HAX1 1#**	GGGCCGGACAGAGACTACAGT	Target sequence for HAX1 knockdown in Huh7 cells
**sh human-HAX1 2#**	GACTCGAGATGAAGATGATGA	Target sequence for HAX1 knockdown in Huh7 cells
**sh mouse-HAX1**	GCAGACACTACGAGACTCAAT	Target sequence for HAX1 knockdown in mice
**sgRNA-HAX1**	AGTACGAGATTTCAATAGCA	Target sequence for HAX1 knockout in HEK293 cells
