# Supplementary material for: Interactome profiling of Crimean-Congo hemorrhagic fever virus glycoproteins
Source: Nat Commun. 2023 Nov 14;14:7365. doi: 10.1038/s41467-023-43206-1 (PMC10646030; doi:10.1038/s41467-023-43206-1)
Supplement: Supplementary file 3 — Reporting Summary [file 41467_2023_43206_MOESM3_ESM.pdf]

nature portfolio

Corresponding author(s): Yun-Jia Ning

Last updated by author(s): Oct 21, 2023

Reporting Summary

Nature Portfolio wishes to improve the reproducibility of the work that we publish. This form provides structure for consistency and transparency in reporting. For further information on Nature Portfolio policies, see our Editorial Policies and the Editorial Policy Checklist.

Statistics

For all statistical analyses, confirm that the following items are present in the figure legend, table legend, main text, or Methods section.

n/a	Confirmed	
☐	☒	The exact sample size (*n*) for each experimental group/condition, given as a discrete number and unit of measurement
☐	☒	A statement on whether measurements were taken from distinct samples or whether the same sample was measured repeatedly
☐	☒	The statistical test(s) used AND whether they are one- or two-sided<br>*Only common tests should be described solely by name; describe more complex techniques in the Methods section.*
☒	☐	A description of all covariates tested
☒	☐	A description of any assumptions or corrections, such as tests of normality and adjustment for multiple comparisons
☐	☒	A full description of the statistical parameters including central tendency (e.g. means) or other basic estimates (e.g. regression coefficient) AND variation (e.g. standard deviation) or associated estimates of uncertainty (e.g. confidence intervals)
☐	☒	For null hypothesis testing, the test statistic (e.g. *F*, *t*, *r*) with confidence intervals, effect sizes, degrees of freedom and *P* value noted<br>*Give P values as exact values whenever suitable.*
☒	☐	For Bayesian analysis, information on the choice of priors and Markov chain Monte Carlo settings
☒	☐	For hierarchical and complex designs, identification of the appropriate level for tests and full reporting of outcomes
☐	☒	Estimates of effect sizes (e.g. Cohen's *d*, Pearson's *r*), indicating how they were calculated

Our web collection on statistics for biologists contains articles on many of the points above.

Software and code

Policy information about availability of computer code

Data collection	All software is publicly or commercially available. See methods section for literature references.<br>To collect the data, we used the following software:<br>Western blot: SuperSignal™ West Pico PLUS Chemiluminescent System<br>RT-qPCR: StepOnePlus Real-Time PCR System<br>Microscopy: Andor Confocal Microscope (Dragonfly 202)<br>Flow cytometry: BD LSRFortessa flow cytometer
Data analysis	All software is publicly or commercially available. To analyse the data, we used the following softwares:<br>Western blot: ImageJ version Fiji (http://imagej.nih.gov/ij)<br>Microscopy: ImageJ version Fiji (http://imagej.nih.gov/ij)<br>RT-qPCR: Microsoft Excel 2016<br>Flow cytometry: FlowJo software (version 7.6)<br>MS: hybrid quadrupole-TOF mass spectrometer (TripleTOF 5600+, SCIEX), MaxQuant (V1.6.2.10)<br>GO analysis: PANTHER (http://www.pantherdb.org/), REVIGO (http://revigo.irb.hr/)<br>PPI network: STRING (https://string-db.org/), Cytoscape (version number: 3.6.1).<br>Statistical analyses: GraphPad Prism 8 (GraphPad Software, San Diego California USA, https://www.graphpad.com/)<br>Schematic drawing: Adobe illustrator cc 2018

For manuscripts utilizing custom algorithms or software that are central to the research but not yet described in published literature, software must be made available to editors and reviewers. We strongly encourage code deposition in a community repository (e.g. GitHub). See the Nature Portfolio guidelines for submitting code & software for further information.

Data

Policy information about availability of data

All manuscripts must include a data availability statement. This statement should provide the following information, where applicable:

- Accession codes, unique identifiers, or web links for publicly available datasets
- A description of any restrictions on data availability
- For clinical datasets or third party data, please ensure that the statement adheres to our policy

The raw MS proteomics data have been deposited to the Science Data Bank (CSTR: 31253.11.SCIENCEDB.10567), and the access link is https://www.scidb.cn/en/s/Rn2INj. Other data are contained within the article.
All the raw data is available through source data file.

Research involving human participants, their data, or biological material

Policy information about studies with human participants or human data. See also policy information about sex, gender (identity/presentation), and sexual orientation and race, ethnicity and racism.

Reporting on sex and gender	N/A
Reporting on race, ethnicity, or other socially relevant groupings	N/A
Population characteristics	N/A
Recruitment	N/A
Ethics oversight	N/A

Note that full information on the approval of the study protocol must also be provided in the manuscript.

Field-specific reporting

Please select the one below that is the best fit for your research. If you are not sure, read the appropriate sections before making your selection.

☒ Life sciences ☐ Behavioural & social sciences ☐ Ecological, evolutionary & environmental sciences

For a reference copy of the document with all sections, see nature.com/documents/nr-reporting-summary-flat.pdf

Life sciences study design

All studies must disclose on these points even when the disclosure is negative.

Sample size	No sample size calculation was performed. All experiments were performed at least in triplicate, which is common practice for molecular biology experiments. In this study, sample size was determined based on the standards for cell experiments attempting to have a minimum of N = 3 biological independent samples and animal experiments attempting to have a minimum of N = 4 biological independent samples with sufficient reproducibility. Quantitative analysis of confocal images included replicates of different field scans per independent sample. This sample size was determined to be sufficient for calculating average and standard error and eventually statistical significance amongst different groups. The details on the sample size were included in each figure legend.
Data exclusions	No data was excluded.
Replication	In this study, each result described in the paper is based on at least three independent biological replicates but very often an experiment is based on more than three experiments. Figure legends indicate the number of independent experiments performed in each analysis.
Randomization	For all the experiments, samples were randomly allocated to experimental and control groups.
Blinding	Blinding was not relevant for this study as no subjective rating (e. gmanual counting of features) was performed. Quantitative measurements were performed by machines and the effects described in qualitative experiments were obvious.

Behavioural & social sciences study design

All studies must disclose on these points even when the disclosure is negative.

Study description	N/A
Research sample	N/A
Sampling strategy	N/A
Data collection	N/A
Timing	N/A
Data exclusions	N/A
Non-participation	N/A
Randomization	N/A

Ecological, evolutionary & environmental sciences study design

All studies must disclose on these points even when the disclosure is negative.

Study description	N/A
Research sample	N/A
Sampling strategy	N/A
Data collection	N/A
Timing and spatial scale	N/A
Data exclusions	N/A
Reproducibility	N/A
Randomization	N/A
Blinding	N/A

Did the study involve field work? ☐ Yes ☐ No

Field work, collection and transport

Field conditions	N/A
Location	N/A
Access & import/export	N/A
Disturbance	N/A

Reporting for specific materials, systems and methods

We require information from authors about some types of materials, experimental systems and methods used in many studies. Here, indicate whether each material, system or method listed is relevant to your study. If you are not sure if a list item applies to your research, read the appropriate section before selecting a response.

Materials & experimental systems

n/a	Involved in the study
☐	☒ Antibodies
☐	☒ Eukaryotic cell lines
☒	☐ Palaeontology and archaeology
☐	☒ Animals and other organisms
☒	☐ Clinical data
☒	☐ Dual use research of concern
☒	☐ Plants

Methods

n/a	Involved in the study
☒	☐ ChIP-seq
☐	☒ Flow cytometry
☒	☐ MRI-based neuroimaging

Antibodies

Antibodies used

Mouse monoclonal antibodies (mAb) against CCHFV Gc and NP and mouse or rabbit polyclonal antibodies (pAb) against CCHFV Gn were prepared in house. Antibodies against S-tag (Sino Biological, Cat#101290-T38, Dilution 1:2000), Strep-tag (Sangon Biotech, Cat#D191106, Dilution 1:2000), HAX1 (Proteintech, Cat#11266-1-AP, Dilution 1:1000), KPNB1 (ABclonal, Cat#A8610, Dilution 1:1000), RPN1 (Abcam, Cat#ab198508, Dilution 1:2000), HADHA (ABclonal, Cat#A13310, Dilution 1:1000), THBS4 (ABclonal, Cat#A16438, Dilution 1:1000), ATP1A1 (ABclonal, Cat#A7878, Dilution 1:1000), COXIV (Proteintech, Cat#11242-1-AP, Dilution 1:1000), GAPDH (Proteintech, Cat#10494-1-AP, Dilution 1:5000), β-actin (Proteintech, Cat#66009-1-Ig, Dilution 1:5000), HRP-labled goat anti-mouse (Proteintech, Cat#SA00001-1, Dilution 1:5000) or anti-rabbit IgG (Proteintech, Cat#SA00001-2, Dilution 1:5000) antibodies, Alexa Fluor-488 goat anti-mouse IgG (Abcam, Cat#ab 150113, Dilution 1:1000), Alexa Fluor-555 goat anti-mouse IgG (Abcam, Cat#ab 150114, Dilution 1:1000), and Alexa Fluor-647 goat anti-rabbit IgG (Abcam, Cat#ab 150079, Dilution 1:1000) were purchased from the indicated manufacturers, respectively.

Validation

S-tag antibody: https://www.sinobiological.com/antibodies/s-tag-101290-t38. Validated by manufacturer for WB and IF.
Strep-tag antibody: https://store.sangon.com/productDetail?productInfo.code=D191106. Validated by manufacturer for WB.
HAX1 antibody: https://www.ptgcn.com/products/HAX1-Antibody-11266-1-AP.htm. Validated by manufacturer for WB, Correct size.
KPNB1 antibody: https://abclonal.com.cn/catalog/A8610. Validated by manufacturer for WB, Correct size, IF: Correct cellar localization.
RPN1 antibody: https://www.abcam.cn/products/primary-antibodies/ribophorin-i-antibody-epr17043b-n-terminal-ab198508.html. Validated by manufacturer for WB, Correct size, IF: Correct cellar localization.
HADHA antibody: https://abclonal.com.cn/catalog/A13310. Validated by manufacturer for WB, Correct size, IF: Correct cellar localization.
THBS4 antibody: https://abclonal.com.cn/catalog/A16438. Validated by manufacturer for WB, Correct size.
ATP1A1 antibody: https://abclonal.com.cn/catalog/A7878. Validated by manufacturer for WB, Correct size.
COXIV antibody: https://www.ptgcn.com/products/COX4I1-Antibody-11242-1-AP.htm. Validated by manufacturer for WB, Correct size, IF: Correct cellar localization.
GAPDH antibody: https://www.ptgcn.com/products/GAPDH-Antibody-10494-1-AP.htm. Validated by manufacturer for WB, Correct size, IF: Correct cellar localization.
β-actin antibody: https://www.ptgcn.com/products/Pan-Actin-Antibody-66009-1-Ig.htm. Validated by manufacturer for WB, Correct size, IF: Correct cellar localization.
HRP-labled goat anti-mouse IgG: https://www.ptgcn.com/products/HRP-conjugated-Affinipure-Goat-Anti-Mouse-IgG-H-L-secondary-antibody.htm. Validated by manufacturer for WB.
HRP-labled goat anti-rabbit IgG: https://www.ptgcn.com/products/HRP-conjugated-Affinipure-Goat-Anti-Rabbit-IgG-H-L-secondary-antibody.htm. Validated by manufacturer for WB.
Alexa Fluor-488 goat anti-mouse IgG: https://www.abcam.cn/products/secondary-antibodies/goat-mouse-igg-hl-alexa-fluor-488-ab150113.html. Validated by manufacturer for IF.
Alexa Fluor-555 goat anti-mouse IgG: https://www.abcam.cn/products/secondary-antibodies/goat-mouse-igg-hl-alexa-fluor-555-ab150114.html. Validated by manufacturer for IF.
Alexa Fluor-647 goat anti-rabbit IgG: https://www.abcam.cn/products/secondary-antibodies/goat-rabbit-igg-hl-alexa-fluor-647-ab150079.html. Validated by manufacturer for IF.

Eukaryotic cell lines

Policy information about cell lines and Sex and Gender in Research

Cell line source(s)	HEK293T, (ATCC, CRL-3216); Huh7, (NVRC, IVCAS 9.005); HEK293, (ATCC, CRL-1573); HeLa, (ATCC, CCL-2).
Authentication	None of these cell lines were authenticated by us.
Mycoplasma contamination	Cell tested negative for mycoplasma.
Commonly misidentified lines (See ICLAC register)	No commonly misidentified cell lines were used.

Palaeontology and Archaeology

Specimen provenance	N/A
Specimen deposition	N/A
Dating methods	N/A

☐ Tick this box to confirm that the raw and calibrated dates are available in the paper or in Supplementary Information.

Ethics oversight	N/A

Note that full information on the approval of the study protocol must also be provided in the manuscript.

Animals and other research organisms

Policy information about studies involving animals; ARRIVE guidelines recommended for reporting animal research, and Sex and Gender in Research

Laboratory animals	The 6-8 week-old female C57BL/6 mice were used.
Wild animals	No wild animals were used in this study.
Reporting on sex	Sex as a variable was not tested in this study.
Field-collected samples	No field-collected samples were used in this study.
Ethics oversight	All animal experiment procedures were approved in advance by the ethics committees of Wuhan Institute of Virology, Chinese Academy of Sciences (approval number: WIVA33202207) .

Note that full information on the approval of the study protocol must also be provided in the manuscript.

Clinical data

Policy information about clinical studies

All manuscripts should comply with the ICMJE guidelines for publication of clinical research and a completed CONSORT checklist must be included with all submissions.

Clinical trial registration	N/A
Study protocol	N/A
Data collection	N/A
Outcomes	N/A

Dual use research of concern

Policy information about dual use research of concern

Hazards

Could the accidental, deliberate or reckless misuse of agents or technologies generated in the work, or the application of information presented in the manuscript, pose a threat to:

No	Yes	
☒	☐	Public health
☒	☐	National security
☒	☐	Crops and/or livestock
☒	☐	Ecosystems
☒	☐	Any other significant area

Experiments of concern

Does the work involve any of these experiments of concern:

No	Yes	
☒	☐	Demonstrate how to render a vaccine ineffective
☒	☐	Confer resistance to therapeutically useful antibiotics or antiviral agents
☒	☐	Enhance the virulence of a pathogen or render a nonpathogen virulent
☒	☐	Increase transmissibility of a pathogen
☒	☐	Alter the host range of a pathogen
☒	☐	Enable evasion of diagnostic/detection modalities
☒	☐	Enable the weaponization of a biological agent or toxin
☒	☐	Any other potentially harmful combination of experiments and agents

Plants

Seed stocks	N/A
Novel plant genotypes	N/A
Authentication	N/A

ChIP-seq

Data deposition

☐ Confirm that both raw and final processed data have been deposited in a public database such as GEO.

☐ Confirm that you have deposited or provided access to graph files (e.g. BED files) for the called peaks.

Data access links<br>*May remain private before publication.*	N/A
Files in database submission	N/A
Genome browser session<br>(e.g. UCSC)	N/A

Methodology

Replicates	N/A
Sequencing depth	N/A
Antibodies	N/A
Peak calling parameters	N/A
Data quality	N/A
Software	N/A

Flow Cytometry

Plots

Confirm that:

☒ The axis labels state the marker and fluorochrome used (e.g. CD4-FITC).

☒ The axis scales are clearly visible. Include numbers along axes only for bottom left plot of group (a 'group' is an analysis of identical markers).

☒ All plots are contour plots with outliers or pseudocolor plots.

☒ A numerical value for number of cells or percentage (with statistics) is provided.

Methodology

Sample preparation	HEK293T cells infected with pseudotyped virus expressing GFP was harvested in PBS.
Instrument	BD LSRFortessa flow cytometer
Software	FlowJo software (version 7.6)
Cell population abundance	A minimum of 3×10^4 total evants were recorded for all samples.
Gating strategy	A preliminary FSC-H vs SSC-H gating was used for all cells and then gated by FITC (EGFP).

☒ Tick this box to confirm that a figure exemplifying the gating strategy is provided in the Supplementary Information.

Magnetic resonance imaging

Experimental design

Design type	N/A
Design specifications	N/A
Behavioral performance measures	N/A

Acquisition

Imaging type(s)	N/A
Field strength	N/A
Sequence & imaging parameters	N/A
Area of acquisition	N/A

Diffusion MRI ☐ Used ☐ Not used

Preprocessing

Preprocessing software	N/A
Normalization	N/A
Normalization template	N/A
Noise and artifact removal	N/A
Volume censoring	N/A

Statistical modeling & inference

Model type and settings	N/A
Effect(s) tested	N/A

Specify type of analysis: ☐ Whole brain ☐ ROI-based ☐ Both

Statistic type for inference (See Eklund et al. 2016)	N/A
Correction	N/A

Models & analysis

n/a	Involved in the study
☒	☐ Functional and/or effective connectivity
☒	☐ Graph analysis
☒	☐ Multivariate modeling or predictive analysis

Functional and/or effective connectivity	*Report the measures of dependence used and the model details (e.g. Pearson correlation, partial correlation, mutual information).*
Graph analysis	*Report the dependent variable and connectivity measure, specifying weighted graph or binarized graph, subject- or group-level, and the global and/or node summaries used (e.g. clustering coefficient, efficiency, etc.).*
Multivariate modeling and predictive analysis	*Specify independent variables, features extraction and dimension reduction, model, training and evaluation metrics.*
